# Discovery and Extrolite Production of Three New Species of *Talaromyces* Belonging to Sections *Helici* and *Purpurei* from Freshwater in Korea

**DOI:** 10.3390/jof7090722

**Published:** 2021-09-03

**Authors:** Thuong T. T. Nguyen, Jens Christian Frisvad, Paul M. Kirk, Hyo Jin Lim, Hyang Burm Lee

**Affiliations:** 1 Environmental Microbiology Lab, Department of Agricultural Biological Chemistry, College of Agriculture and Life Sciences, Chonnam National University, Gwangju 61186, Korea; ngthuongthuong@gmail.com (T.T.T.N.); hyojinzzanggu95@naver.com (H.J.L.); 2 Department of Biotechnology and Biomedicine, Technical University of Denmark, 2800 Kongens Lyngby, Denmark; jcf@bio.dtu.dk; 3 Biodiversity Informatics and Spatial Analysis, Jodrell Laboratory, Royal Botanic Gardens Kew, Surrey TW9 3DS, UK; P.Kirk@kew.org

**Keywords:** three new taxa, *Trichocomaceae*, morphology, phylogeny, taxonomy

## Abstract

Three novel fungal species, *Talaromyces* *gwangjuensis*, *T. koreana*, and *T. teleomorpha* were found in Korea during an investigation of fungi in freshwater. The new species are described here using morphological characters, a multi-gene phylogenetic analysis of the ITS, *BenA*, *CaM*, *RPB2* regions, and extrolite data. *Talaromyces* *gwangjuensis* is characterized by restricted growth on CYA, YES, monoverticillate and biverticillate conidiophores, and globose smooth-walled conidia. *Talaromyces koreana* is characterized by fast growth on MEA, biverticillate conidiophores, or sometimes with additional branches and the production of acid on CREA. *Talaromyces teleomorpha* is characterized by producing creamish-white or yellow ascomata on OA and MEA, restricted growth on CREA, and no asexual morph observed in the culture. A phylogenetic analysis of the ITS, *BenA*, *CaM*, and *RPB2* sequences showed that the three new taxa form distinct monophyletic clades. Detailed descriptions, illustrations, and phylogenetic trees are provided.

## 1. Introduction

The genus *Talaromyces* was established by Benjamin (1955) [1] for a teleomorph of *Penicillium* with *Talaromyces vermiculatus* (=*T. flavus*) as the type species. These species are characterized by cleistothecial or gymnothecial ascomata, unitunicate eight-spored asci, and unicellular ascospores with or without equatorial crests. The anamorphs have predominantly biverticillate or rarely terverticillate conidiophores with acerose phialides and narrow collulum [2,3]. In 2011, Samson et al. [2] transferred all accepted species of *Penicillium* subgen. *Biverticillium* to *Talaromyces* on the basis of a two-gene phylogeny. Subsequently, Yilmaz et al. [3] studied the taxonomy of *Talaromyces* in detail using the polyphasic species concept. On the basis of multigene phylogeny, morphology, and physiology, Yilmaz et al. [3] placed 88 accepted species in seven well-defined sections, namely, *Bacillispori*, *Helici*, *Islandici*, *Purpurei*, *Subinflati*, *Talaromyces*, and *Trachyspermi*. However, the lists are rapidly increasing with many new *Talaromyces* species recently described from all over the world and added to sections *Helici*, *Islandici*, *Purpurei*, *Subinflati*, *Talaromyces*, and *Trachyspermi* [4,5,6,7,8,9,10,11,12,13,14,15,16,17,18,19,20,21,22,23,24,25,26,27]. To date, 171 species have been reported in the genus *Talaromyces* [27], of which only three species: *Talaromyces angelicae*, *Talaromyces cnidii*, and *Talaromyces halophytorum* were reported from Korea [28,29]. Recently, a new section *Tenues* was proposed [26]. *Talaromyces* contains species that play an important role in agriculture and biotechnology. *Talaromyces rugulosus* (Basionym: *Penicillum rugulosum*) produces β-rutinosidase and phosphatase [30,31], *T. pinophilus* (Basionym: *Penicillium pinophilum*) produces endoglucanase and cellulase [32], and *T. funiculosus* (Basionym: *Penicillium funiculosum*) produces cellulases [33]. *Talaromyces purpureogenus* can produce extracellular enzymes and red pigment and also produces mycotoxin such as rubratoxin A and B and luteoskyrin [34]. Additionally, red pigments produced in large amounts by *T. atroroseus* can be used as colorants in the food industry [35]. Furthermore, the ability to produce various important compounds makes them candidates for the biocontrol of soilborne fungal pathogens such as an antagonists of *T. flavus* against *Verticillium* spp., *Rhizoctonia solani*, and *Sclerotinia sclerotiorum* [36,37,38,39,40]. In addition, some species are medically important, such as *T. wortmannii*, which can produce compound C that was found to be an effective antimicrobial against *Propionibacterium acnes* and had anti-inflammatory properties and, thus, represents alternative treatments for antibiotic or anti-inflammatory therapy for acne [41]. *Talaromyces marneffei* (Basionym: *Penicillium marneffei*) causes a fatal mycosis in immunocompromised individuals [42,43].

Section *Helici* was proposed by Yilmaz et al. [3] with seven *Talaromyces* species divided into two clades: a main clade containing *T. helicus*, *T. boninensis*, and *T. varians* and a second clade containing *T. cinnabarinus*, *T. aerugineus*, *T. bohemicus*, and *T. ryukyuensis*. The *Talaromyces* species included in this section are characterized by producing biverticillate conidiophores occasionally consisting of solitary phialides with stipes generally pigmented, yellowish-brown, or dark green reversed on CYA; grown at 37 °C, and the absence of acid production on CREA [3]. Section *Helici* currently includes 13 species [27].

Section *Purpurei* was proposed by Stolk and Samson [44] to accommodate species that produce synnemata after two to three weeks of incubation, with the exception of *T. rademirici*, *T. purpureus*, and *T. ptychoconidium*. The species in this section generally do not grow or grow poorly on creatine sucrose agar (CREA), and grow restrictedly on Czapek yeast extract agar (CYA) and yeast extract sucrose agar (YES) and slightly faster on malt extract agar (MEA) [3]. Ten species were accepted in the section *Purpurei*: *T. cecidicola*, *T. chloroloma*, *T. coalescens*, *T. dendriticus*, *T. pseudostromaticus*, *T. pittii*, *T. purpureus*, *T. ptychoconidium*, *T. rademirici*, and *T. ramulosus* [3], but it currently contains 12 species [27].

Freshwater fungi are an ubiquitous and diverse group of organisms and play an important role in ecological systems [45]. Hawksworth [46] estimated that there are approximately 1.5 million fungal species on Earth. However, an updated estimate of the number of fungal species is between 2.2 and 3.8 million [47]. Of the ca. 150,000 known sepecies, only around 3000 have been reported from aquatic habitats [48], with more than 600 species of ascomycetes reported in freshwater [49]. Thus, a large number of species are still waiting to be discovered and described in freshwater habitats.

Up to now, only a few freshwater fungi, especially genus *Talaromyces*, have been reported in Korea. The purpose of this study was to expand the present knowledge of these fungal taxa in Korea. Here, we describe and illustrate three new *Talaromyces* species from freshwater habitats in Korea.

## 2. Materials and Methods

### 2.1. Sampling and Isolation

In January and May 2017, freshwater samples were collected from the Wonhyo Valley located at Mudeung Mt., Gwangju, and Jukrim Reservoir located in Yeosu, Korea. These samples were transported to the laboratory in sterile 50-mL conical tubes and stored at 4 °C pending examination. Before culture preparation, all samples were diluted with sterile distilled water to reduce the density and improve strain recovery. Briefly, each sample was shaken for 15 min at room temperature, and a 100-μL aliquot of each sample was mixed with 9 mL of sterile distilled water. Then, serial dilutions of the mixture (from 10^−1^ to 10^−4^) were made. A 100-μL aliquot of each dilution was spread on potato dextrose agar (PDA: 39 g of potato dextrose agar in 1 L of deionized water; Becton, Dickinson, and Co., Sparks, MD, USA) supplemented with the antibiotic streptomycin (final concentration, 50 ppm; Sigma-Aldrich, St. Louis, MO, USA). The petri plates were incubated at 25 °C for 5–10 days. Pure isolates were obtained by selecting individual colonies of varied morphologies, transferring them to PDA plates, and subculturing until pure cultures were obtained. Ex-type living cultures were deposited in the Environmental Microbiology Laboratory Fungarium, Chonnam National University (CNUFC), Gwangju, Korea. Dried cultures were deposited in the Herbarium Chonnam National University, Gwangju, Korea.

### 2.2. Morphology

The strains were three-point inoculated onto Czapek yeast autolysate agar (CYA), malt extract agar (MEA), yeast extract sucrose agar (YES), oatmeal agar (OA), dichloran 18% glycerol (DG18) agar, CYA supplemented with 5% NaCl (CYAS), and creatine sucrose agar (CREA). All petri dishes were incubated at 20, 25, 30, 35, 37, and 40 °C for 7 days. Medium preparation and inoculation were performed according to the methods reported by Yilmaz et al. [3]. Colony characters were recorded after 7 days. Lactic acid (60%) was used as the mount fluid, and 96% ethanol was used to remove excess conidia. The Olympus BX51 microscope with differential interference contrast optics (Olympus, Tokyo, Japan) was used to obtain digital images. For scanning electron microscopy (SEM), the samples were performed as described previously by Nguyen et al. [50].

### 2.3. DNA Extraction, PCR, and Sequencing

The fungal isolates were cultured on PDA overlaid with cellophane at 25 °C for 5–7 days. Genomic DNA was extracted using the Solg^TM^ Genomic DNA Preparation Kit (Solgent Co. Ltd., Daejeon, Korea). The ITS region was amplified using the primer pairs ITS 1 and ITS 4 [51]. The beta-tubulin (*BenA*) was amplified using the primer pairs T10 and Bt2b [52]. The calmodulin (*CaM*) gene was amplified using the primer pairs CMD5/CMD6 and CF1/CF4 [53,54]. To amplify the *RPB2* gene region, the primer pairs RPB2-5F and RPB2-7cR were used [55]. PCR amplification was performed according to the conditions described by Yilmaz et al. [3] and Houbraken and Samson [56]. The PCR products were purified with the Accuprep PCR Purification Kit (Bioneer Corp., Daejeon, Korea). Sequencing was performed using the same PCR primers and run on the ABI PRISM 3730XL Genetic Analyzer (Applied Biosystems, Foster City, CA, USA).

### 2.4. Molecular Analysis

Each generated sequence was checked for the presence of ambiguous bases and assembled using the Lasergene SeqMan program from DNASTAR, Inc. (Madison, WI, USA). Edited sequences were blasted against the NCBI GenBank nucleotide database (https://blast.ncbi.nlm.nih.gov/Blast.cgi; 2 January 2021) to search for the closest relatives. The sequences of all the accepted *Talaromyces* species were retrieved from GenBank. The sequences were aligned using MAFFT (https://mafft.cbrc.jp/alignment/server; 9 March 2021) [57], and the resulting alignment was trimmed using trimAl [58] and subsequently combined with MEGA 7 [59]. The data were converted from a FASTA format to nexus and phylip formats using the online tool Alignment Transformation Environment (https://sing.ei.uvigo.es/ALTER/; 9 March 2021) [60]. Phylogenetic reconstructions by maximum likelihood (ML) were carried out using RAxML-HPC2 on XSEDE on the online CIPRES Portal (https://www.phylo.org/portal2; 9 March 2021) with 1000 bootstrap replicates and the GTRGAMMA model of nucleotide substitution. A Bayesian inference analysis was performed with MrBayes 3.2.2 [61] using a Markov Chain Monte Carlo (MCMC) algorithm. The sample frequency was set to 100, and the first 25% of trees were removed as burn-in. The trees were visualized using FigTree v. 1.3.1 [62]. Support values were provided at the branches (ML bootstrap support (BS) and BI posterior probability (PP)). *Talaromyces tenuis* CBS 141840 was chosen as the outgroup in the sections *Helici* and *Purpurei* phylogenies. *Trichocoma paradoxa* CBS 788.83 was the outgroup for the combined phylogeny of the species from *Talaromyces*. The newly obtained sequences were deposited in the GenBank database under the accession numbers provided in Table 1.

### 2.5. Extrolite Analysis

Extrolites were extracted from *Talaromyces* strains after growing on CYA, YES, and MEA for 7–10 days at 25 °C. The extracts were prepared and analyzed as previously described by Frisvad and Thrane [63], Nielsen et al. [64], and Houbraken et al. [65].

## 3. Results

### 3.1. Phylogenetic Analysis

**Figure 1:**
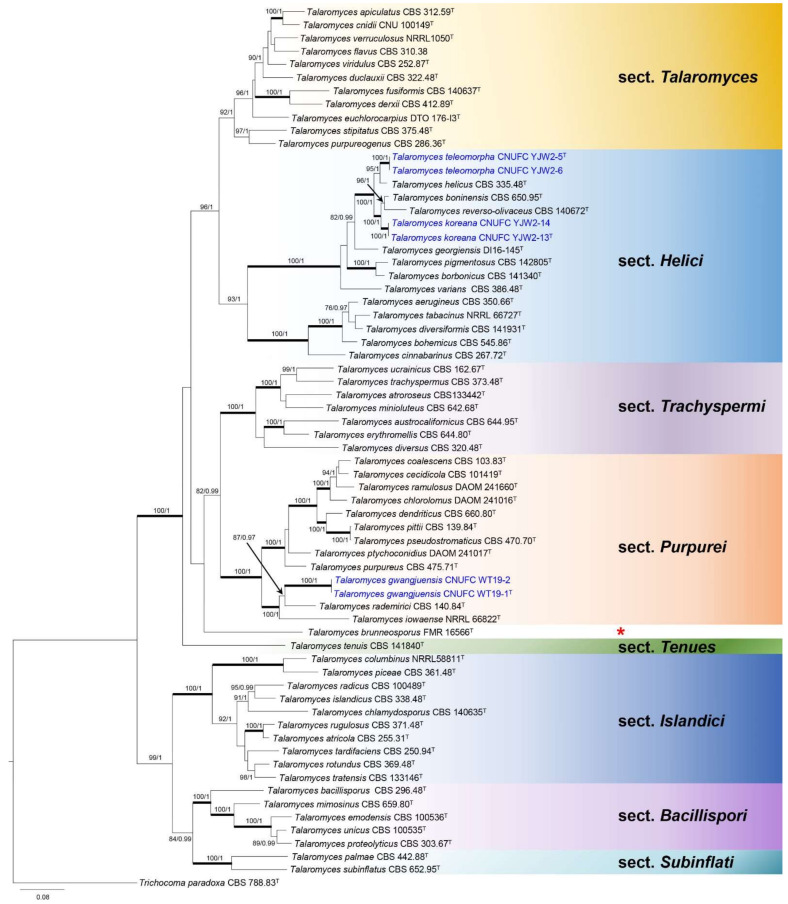
Phylogram generated from the Maximum Likelihood (RAxML) analysis based on the combined ITS, *BenA*, *CaM*, and *RPB2* sequences data of *Talaromyces*. The red asterisk represents a separate lineage which is not assigned yet. The branches with values = 100% ML BS and 1 PP are highlighted by thickened branches. The branches with values ≥70% ML BS and ≥0.95 PP indicated above or below branches. *Trichocoma paradoxa* CBS 788.83 was the group was used as the outgroup. The newly generated sequences are indicated in blue. ^T^ = ex-type.

**Figure 2:**
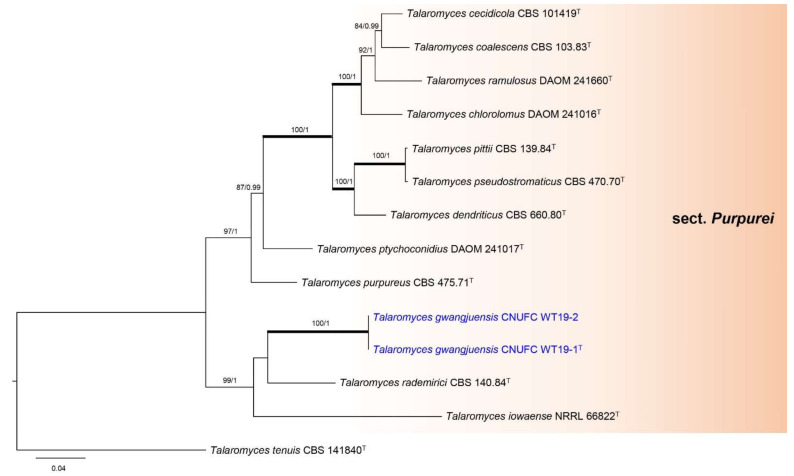
Phylogram generated from the Maximum Likelihood (RAxML) analysis based on the combined ITS, *BenA*, *CaM*, and *RPB2* sequences data for species classified in *Talaromyces* section *Purpurei*. The branches with values = 100% ML BS and 1 PP are highlighted by thickened branches. The branches with values ≥70% ML BS and ≥0.95 PP indicated above or below branches. *Talaromyces tenuis* CBS 141840 was used as the outgroup. The newly generated sequences are indicated in blue. ^T^ = ex-type.

**Figure 3:**
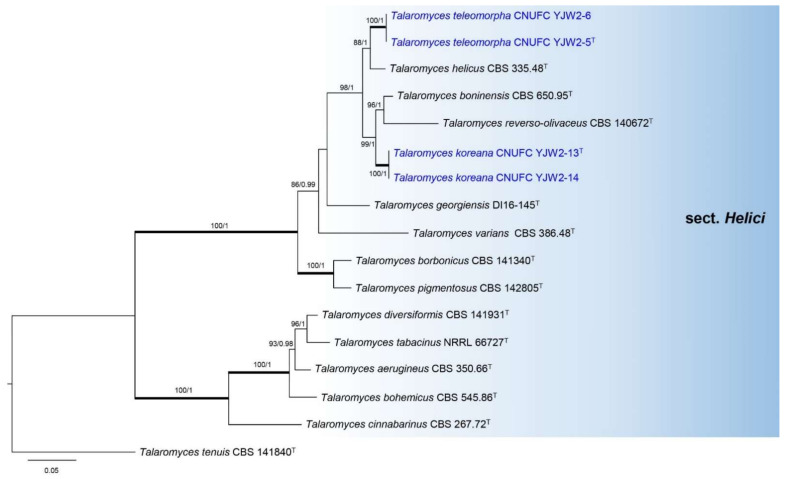
Phylogram generated from the Maximum Likelihood (RAxML) analysis based on combined the ITS, *BenA*, *CaM*, and *RPB2* sequence data for the species classified in *Talaromyces* section *Helici*. The branches with values = 100% ML BS and 1 PP are highlighted by thickened branches. The branches with values ≥70% ML BS and ≥0.95 PP indicated above or below branches. *Talaromyces tenuis* CBS 141840 was used as the outgroup. The newly generated sequences are indicated in blue. ^T^ = ex-type.

Phylogenetic relationships within *Talaromyces* were studied using a concatenated dataset of four loci (ITS, *BenA*, *CaM*, and *RPB2*) (Figure 1). The multigene analysis contained 67 taxa, including *Trichocoma paradoxa* CBS 788.83 as the outgroup taxon. The concatenated alignment consisted of 2407 characters (including alignment gaps): 425, 443, 687, and 852 characters used in the ITS, *BenA*, *CaM*, and *RPB2*, respectively. Eight main lineages are present within *Talaromyces*, which agrees with the sectional classification by Yilmaz et al. [3] and Sun et al. [26]. In the phylogenetic analysis, a small clade containing *T. brunneosporus* highlighted by asterisk could not be assigned to any known sections (Figure 1). *Talaromyces gwangjuensis*, *T. koreana*, and *T. teleomorpha* belong to sections *Purpurei* and *Helici*, according to our multigene analysis (Figure 1). In section *Purpurei*, *T. gwangjuensis* clustered close to but separated from *T. rademirici* in the single (*BenA*, *RPB2*, and ITS) and combined phylogenies (Figure 2 and Figures S1–S3). *Talaromyces teleomorpha* is close to *T. helicus* in *BenA*, ITS, and combined phylogenies (Figure 3, Figures S4 and S5) but placed among *T. helicus*, *T. koreana*, *T. reverso*-*olivaceus*, and *T. boninensis* in the *CaM* and *RPB2* phylogenies (Figures S6 and S7). *Talaromyces koreana* was found to be related to *T. reverso*-*olivaceus* and *T. boninensis* in *BenA*, *CaM*, *RPB2*, and the combined phylogenies (Figure 3, Figures S4, S6, and S7). In the ITS phylogenetic analysis, *T. koreana* was close to only *T. boninensis* (Figure S5).

### 3.2. Taxonomy

*Talaromycesgwangjuensis* Hyang B. Lee & T.T.T. Nguyen sp. nov.

**Figure 4:**
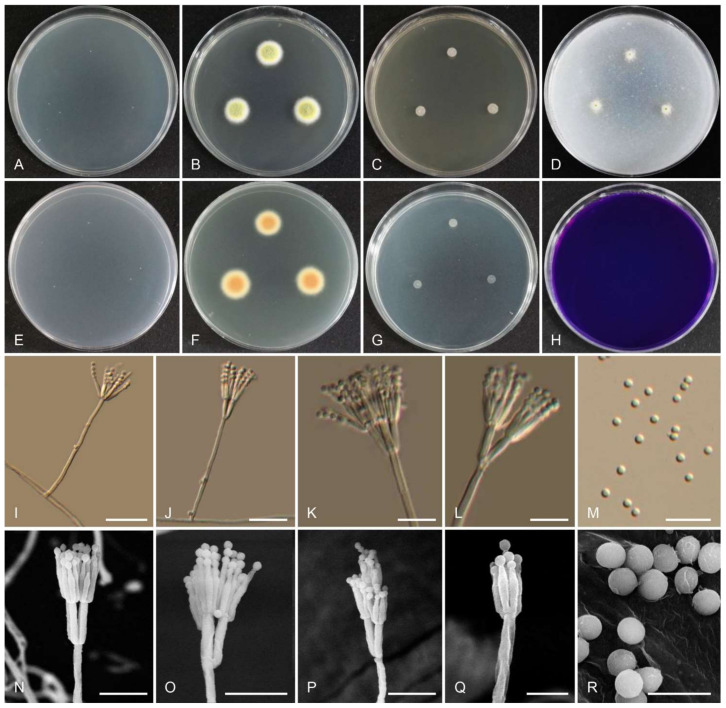
Morphology of *Talaromyces gwangjuensis* CNUFC WT19-1. (**A**,**E**) Colonies on Czapeck yeast autolysate agar (CYA). (**B**,**F**) Malt extract agar (MEA). (**C**) Yeast extract sucrose agar (YES). (**D**) Oatmeal agar (OA). (**G**) Dichloran 18% glycerol agar (DG 18). (**H**) Creatine sucrose agar (CREA). ((**A**–**D**,**G**,**H**) Obverse view and (**E**,**F**) reverse view). (**I**–**L**,**N**–**Q**) Conidiophores. (**M**,**R**) Conidia. ((**I**–**M**) LM and (**N**–**R**) SEM). Scale bars: (**I**–**M**) = 20 μm, (**N**–**Q**) = 10 μm, and (**R**) = 5 μm.

Index Fungorum: IF554801 (Figure 4 and Table 2).

Etymology: Referring to the name of the site where freshwater sample was obtained.

Type specimen: REPUBLIC OF KOREA, Jeonnam Province, Wonhyo Valley located at Mudeung Mt., Gwangju (35°9′1.18″ N, 126°59′24.62″ E) from a freshwater sample, 3 January 2017, H.B. Lee (holotype CNUFC HT19191; ex-type culture CNUFC WT19-1).

Colony diam, 7 d (mm): CYA 25 °C < 1 mm, CYA 20 °C no growth; CYA 30 °C no growth; CYA 37 °C no growth; MEA 25 °C 13–15; YES 25 °C 3–5; OA 25 °C 6–7; CREA 25 °C no growth; CYAS 25 °C no growth; DG18 25 °C 2–4.

Colony characters: CYA 25 °C, 7 d: Colonies low, plane; margins low, entire (<1 mm); mycelia white; sporulation absent; soluble pigments absent; exudates absent; reverse white. MEA 25 °C, 7 d: Colonies strong raised at the center; sporulating central area is dull green, yellow towards the edge; exudate absent; soluble pigments absent; reverse brown-orange center, light yellow near margin. YES 25 °C, 7 d: Sporulation absent, mycelium white; exudate absent; soluble pigments absent; reverse white. OA 25 °C, 7 d: Colony surface velutinous; dull green when sporulating; reverse white; soluble pigments absent; exudates absent. CREA 25 °C, 7 d: No growth. DG18 25 °C, 7 d: No sporulation, mycelium white.

Micromorphology: Sclerotia absent. Conidiophores 39–174 × 1.5–3 µm, biverticillate and monoverticillate. Metulae 2–6, 6–10 × 1.5–2.5 µm. Phialides acerose-shaped, 3–8 per metula, 5.5–10 × 1.5–2 µm. Conidia globose, 1.5–2.0 µm, smooth-walled, conidial chains. Ascomata not observed.

Extrolites: *T. gwangjuensis* (the ex-type strain) produced austin, austinol (and other austins), mitorubrin, mitorubrinol, mitorubrinol acetate, mitorubrinic acid, and a purpactin.

Notes: *Talaromyces gwangjuensis* nested together with *T. rademirici*. However, *T. gwangjuensis* differs morphologically from *T. rademirici*, as it forms smaller colonies on Czapek yeast autolysate agar and yeast extract sucrose agar at 25 °C, and the number of phialides per metula and metulae are larger than those of *T. rademirici*. Furthermore, *T. gwangjuensis* produces globose conidia in contrast with the ellipsoid conidia of *T. rademirici. Talaromyces rademirici* grew at 37 °C, whereas *T. gwangjuensis* did not.

Additional material examined: REPUBLIC OF KOREA, Jeonnam Province, Wonhyo Valley located at Mudeung Mt., Gwangju (35°9′1.18″ N, 126°59′24.62″ E) from a freshwater sample, 4 January 2017, H.B. Lee (culture CNUFC WT19-2).

*Talaromyces koreana* Hyang B. Lee sp. nov.

**Figure 5:**
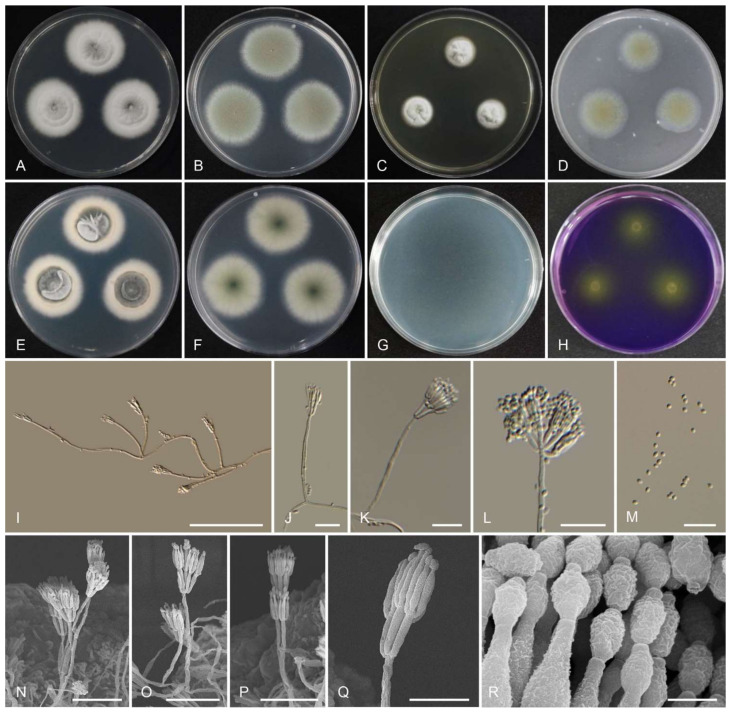
Morphology of *Talaromyces koreana* CNUFC YJW2-13. (**A**,**E**) Colonies on Czapek yeast autolysate agar (CYA). (**B**,**F**) Malt extract agar (MEA). (**C**) Yeast extract sucrose agar (YES). (**D**) Oatmeal agar (OA). (**G**) Dichloran 18% glycerol agar (DG18). (**H**) Creatine sucrose agar (CREA). ((**A**–**D**,**G**,**H**) Obverse view and (**E**,**F**) reverse view). (**I**–**L**,**N**–**Q**) Conidiophores. (**M**,**R**) Conidia. ((**I**–**M**) LM and (**N**–**R**) SEM). Scale bars: (**I**) = 100 µm, (**J**–**L**) = 20 µm, (**M**,**Q**) = 10 µm, (**N**–**P**) = 25 µm, and (**R**) = 2 µm.

Index Fungorum: IF554802 (Figure 5 and Table 3).

Etymology: Referring to the country from which the species was first isolated (Korea).

Type specimen: REPUBLIC OF KOREA, Jeonnam Province, Jukrim reservoir located in Yeosu (34°45′37.72″ N, 127°37′43.46″ E) from a freshwater sample, 26 May 2017, H.B. Lee (CNUFC HT19213 holotype; ex-type culture CNUFC YJW2-13).

Colony diam, 7 d (mm): CYA 25 °C 25–28, CYA 20 °C 15–16, CYA 30 °C 28–31; CYA 37 °C 17–19; MEA 25 °C 41–45; YES 25 °C 21–24; OA 25 °C 36–39; CREA 25 °C 15–18; CYAS 25 °C no growth; DG18 25 °C no growth.

Colony characters: CYA 25 °C, 7 d: Colonies sulcate, raised at the center; margins entire, mycelia slightly murky white; texture floccose; reverse greyish green at the center fading into ivory. MEA 25 °C, 7 d: Colonies low, plane; mycelia white; reverse beige. YES 25 °C, 7 d: Colonies irregularly deep sulcate, raised at the center; margins low, plane, entire (2.5–3 mm); mycelia white; texture floccose; reverse deep olive green. OA 25 °C, 7 d: Colonies low, plane; margins plane, entire (2.5–3 mm); mycelia white; texture velvety; reverse ivory to white. CREA 25 °C, 7 d: Acid production.

Micromorphology: Sclerotia absent. Conidiophores biverticillate, sometimes with additional branches; stipes smooth, 15–194 × 2–4 μm, branches 6–17 × 2–3 μm. Metulae acerose, two to seven, 7.5–16 × 2–3 μm. Phialides acerose, two to seven per metula, 5.5–15 × 2–3 μm. Conidia ellipsoidal to fusiform, finely roughed, 2–3.5 × 1.5–2.5 μm. Ascomata not observed.

Extrolites: Cycloleucomelone, gregatin A, and purpactin A were detected in the ex-type strain of *T. koreana*.

Notes: *Talaromyces koreana* belongs to section *Helici* and is phylogenetically related to *T. boninensis* and *T. reverso-olivaceus. Talaromyces koreana* differs from *T. boninensis* and *T. reverso-olivaceus* by having a higher number of phialides per metula. *Talaromyces koreana* produces smaller conidia than those of *T. boninensis* and *T. reverso-olivaceus*. The maximum colony diameter reported for the species of *T. boninensis* and *T. reverso-olivaceus* are 30 and 34–37 mm when cultivated on MEA at 25 °C in 7 days, while *T. koreana* is 41–45 mm.

Material examined: REPUBLIC OF KOREA, Jeonnam Province, Jukrim reservoir located in Yeosu (34°45′37.72″ N, 127°37′43.46″ E) from a freshwater sample, 27 May 2017, H.B. Lee (culture CNUFC YJW2-14).

*Talaromyces teleomorpha* Hyang B. Lee, Frisvad, P.M. Kirk, H.J. Lim & T.T.T. Nguyen sp. nov.

**Figure 6:**
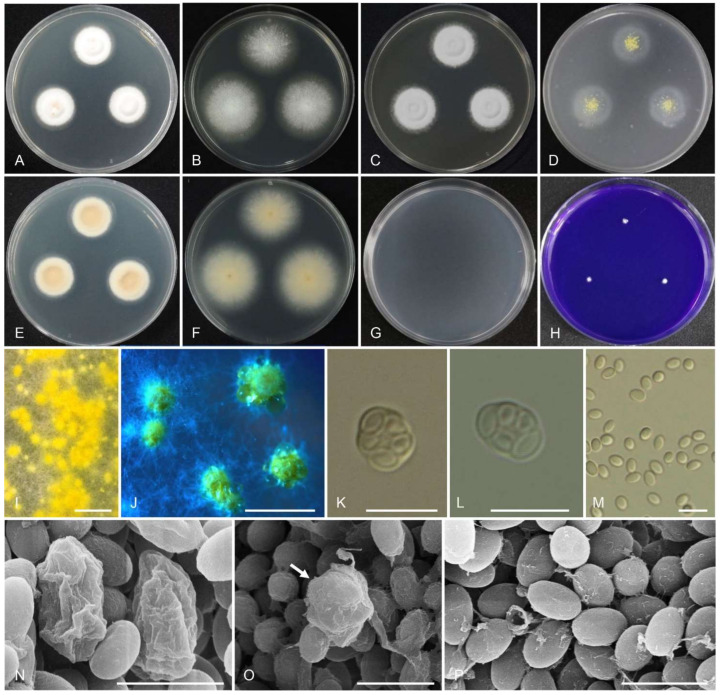
Morphology of *Talaromyces teleomorpha* CNUFC YJW2-5. (**A**,**E**) Colonies on Czapek yeast autolysate agar (CYA). (**B**,**F**) Malt extract agar (MEA). (**C**) Yeast extract sucrose agar (YES). (**D**) Oatmeal agar (OA). (**G**) Dichloran 18% glycerol agar (DG18). (**H**) Creatine sucrose agar (CREA). ((**A**–**D**,**G**,**H**) Obverse view and (**E**,**F**) reverse view). (**I**,**J**) Ascomata. (**K**–**P**) Asci and ascospores. ((**I**,**J**) Stereomicroscope, (**K**–**M**) LM and (**N**–**P**) SEM). Scale bars: (**I**,**J**) = 1 mm, (**K**–**M**) = 10 µm, and (**N**–**P**) = 5 µm.

Index Fungorum: IF554803 (Figure 6 and Table 4).

Etymology: Referring to the teleomorphic stage.

Type specimen: REPUBLIC OF KOREA, Jeonnam Province, Jukrim reservoir located in Yeosu (34°45′37.72″ N, 127°37′43.46″ E) from a freshwater sample, 26 May 2017, H.B. Lee (CNUFC HT19251 holotype; ex-type culture: CNUFC YJW2-5).

Colony diam, 7 d (mm): CYA 25 °C 26–29; CYA 20 °C 15–16; CYA 30 °C 34–36; CYA 37 °C 15–20; MEA 25 °C 45–48; YES 25 °C 29–33; OA 25 °C 32–34; CREA 25 °C 1–3; CYAS 25 °C no growth; DG18 25 °C no growth. 

Colony characters: CYA 25 °C, 7 d: Colonies raised at the center, slightly sulcate; margins low, plane, entire (3 mm); mycelia white to light yellow; reverse ivory to light yellow, slightly sunken at the center. MEA 25 °C, 7 d: colonies low, plane; mycelia white to light yellow, hyaline; reverse light orange at the center. YES 25 °C, 7 d: Colonies raised at the center, sulcate; margins low; mycelia white; reverse pale orange. OA 25 °C, 7 d: Colonies low, plane; mycelia white to light yellow, hyaline, smooth or rough, studded. CREA 25 °C, 7 d: Acid production absent.

Micromorphology: Ascomata maturing within 1 week on OA and MEA at 20–35 °C, abundant, creamish-white to yellow to reddish after long time, usually globose to subglobose, 200–800 μm. Asci ellipsoidal, globose to subglobose, (5.5–)6.5–9 × (4.5–)6–7 μm. Ascospores ellipsoidal, smooth, 3–4 × 2–3 μm.

Notes: *Talaromyces teleomorpha* can be distinguished easily from the closely related species *T. helicus* by growing rapidly on CYA, YES, and MEA at 25 °C in 7 days. Ascomata size of *T. helicus* are smaller than in *T. teleomorpha. Talaromyces helicus* does not grow on CREA, whereas *T. teleomorpha* can grow on this medium. In addition, *T. teleomorpha* does not produce the asexual morph, which is present in *T. helicus*. 

Extrolites: *Talaromyces* *teleomorpha* produced helicusins formerly found in *Talaromyces helicus*.

Material examined: REPUBLIC OF KOREA, Jeonnam Province, Jukrim reservoir located in Yeosu (34°45′37.72″ N, 127°37′43.46″ E) from a freshwater sample, 27 May 2017, H.B. Lee (Culture CNUFC YJW2-6).

## 4. Discussion

During a survey of fungi from a freshwater niche in Korea, three novel species were identified, namely *Talaromyces gwangjuensis*, *T. koreana*, and *T. teleomorpha*.

In our phylogenetic analysis, *Talaromyces gwangjuensis* was classified in section *Purpurei*. This species is closely related to *T. rademirici*, which also has both monoverticillate and biverticillate conidiophores and do not grow on CREA. However, *Talaromyces gwangjuensis* has more restricted colonies on YES and CYA and larger numbers of metulae and phialides. Growth on CYA at 37 °C and the conidial shape and size on MEA at 25 °C can be easily used to distinguish between *T. gwangjuensis* and *T. rademirici. Talaromyces rademirici* grows faster on CYA at all temperatures (CYA at 25 °C, 5–6; CYA at 30 °C, 5–7; CYA at 37 °C, 3), whereas *Talaromyces gwangjuensis* was unable to grow on CYA at 37 °C. Some species in this section have been reported to not grow on CYA at 37 °C, including *T. pittii* and *T. purpureus* [3]; however, *T. pittii* and *T. purpureus* produce ellipsoidal and subglobose to ellipsoidal conidia compared with *T. gwangjuensis* that produces globose conidia.

*Talaromyces koreana* and *T. teleomorpha* belong to the section *Helici*, which was established by Yilmaz et al. [3]. The species in the section was not found to produce acid on CREA medium [3]. However, recent studies showed that *T. georgiensis* and *T. borbonicus* could produce acid on the medium [12,20]. In the present study, *T. koreana* was also found to produce acid on the medium. The results suggest that the ability to produce acid on CREA may not usually a key character to distinguish this section. It is a common character for the species in the section *Helici* to be able to grow at 37 °C [3]. Our results are the same as previous studies [3]. Interestingly, we found that *T. koreana* could grow at 40 °C on MEA media (10–13 mm after 7 days), while not on other media. Our findings showed that the medium composition might influence the maximum growth of fungi.

*Talaromyces teleomorpha* is closely related to *T. helicus*. However, *T. helicus* produces both asexual and sexual morphs, whereas the asexual morph is not observed in *T. teleomorpha* [3]. Especially, *T. teleomorpha* can grow on CREA, while *T. helicus* is unable to grow on this medium [3].

Although ITS is the barcoding marker for fungi [66], this locus is not sufficient to differentiate all *Talaromyces* species. Yilmaz et al. [3] proposed using *BenA* as a secondary molecular marker. In this study, *T. gwangjuensis*, *T. koreana*, and *T. teleomorpha* could be separated via each single gene phylogram. Recently, *T. brunneosporus* was described as a new species discovered from honey in Spain [24]. It was assigned to section *Purpurei* using the ITS, *BenA*, *CaM*, and *RPB2* concatenated dataset. The comparison of ITS, *BenA*, *CaM*, and *RPB2* sequences deposited in GenBank indicated that this species could not be assigned to any known section based on our phylogenetic analyses (Figure 1). In each single gene phylogeny (ITS, *BenA*, *CaM*, and *RPB2*), *T. brunneosporus* also formed a separate lineage (data not shown). More strains are essential to confirm the taxonomic position of *T. brunneosporus*.

Some members from the genus *Talaromyces* are of great interest to the biotechnology industry in medial and food mycology because of their ability to produce a wide range of metabolites [3]. The species of section *Purpurei* produce various extrolite profiles. For example, *T. cecidicola* produces apiculides, pentacecilides, and thailandolides. *Talaromyces coalescens*, *T. dendriticus*, and *T. purpurogenus* share productions of penicillides, purpactins, and vermixocins. On the other hand, *T. purpurogenus* and *T. pseudostromaticus* produce the extrolite mitorubin. Some *Talaromyces* species produce mycotoxins such as botryodiplodin by *T. coalescens*, rugulovasine and luteoskyrin by *T. purpurogenus*, rubratoxins by *T. purpurogenus* and *T. dendriticus*, and secalonic acids D and F by *T. pseudostromaticus. Talaromyces gwangjuensis*, described in this study, produces austin, austinol, mitorubrin, mitorubrinol, mitorubrinol acetate, mitorubrinic acid, and a purpactin without any production of mycotoxins. Some secondary metabolites were found in the section *Helici*, such as alternariol, bacillisporin, and helicusins produced by *T. helicus* [3,67]. *Talaromyces reverso*-*olivaceus* produced rugulovasine A [5], while talaroderxines is produced by *T. boninensis* [3]. In this study, *T. koreana* produced cycloleucomelone, gregatin A, and purpactin A. *Talaromyces teleomorpha* also produced helicusins, as described by Yoshida et al. [67].

*Talaromyces* species are geographically distributed in many kinds of substrates. The species of section *Helici* have been reported to be isolated from soil, cotton yarn, debris, clinical sources, indoor environments, and biomass of *Arundo donax* [3,5,12,15,20]. The species of section *Purpurei* have been reported to be isolated from the air, wasp insect galls, *Eucalyptus*, *Protea repens* infructescence, and other substrates such as apples [3,17,68,69,70,71]. In this study, we isolated three novel species from freshwater. As far as we know, only species belonging to section *Talaromyces* were reported from water [22,72,73,74]. It is interesting to note that *Talaromyces gwangjuensis*, *T. koreana*, and *T. teleomorpha* were the first species in the sections *Purpurei* and *Helici* isolated from freshwater. Our studies expanded our knowledge on the substrates where *Talaromyces* species can occur. Further studies are needed for a better understanding of the ecological roles of these species.

## Figures and Tables

**Table 1:** Accession numbers for the fungal strains used for the phylogenetic analysis.

Taxon Name	Strain No.	GenBank Accession No.				References
		ITS	*BenA*	*CaM*	*RPB2*	
*T. aerugineus*	CBS 350.66 ^T^	AY753346	KJ865736	KJ885285	JN121502	[3]
*T. apiculatus*	CBS 312.59 ^T^	JN899375	KF741916	KF741950	KM023287	[3]
*T. atricola*	CBS 255.31 ^T^	KF984859	KF984566	KF984719	KF984948	[3]
*T. atroroseus*	CBS 133442 ^T^	KF114747	KF114789	KJ775418	KM023288	[3]
*T. austrocalifornicus*	CBS 644.95 ^T^	JN899357	KJ865732	KJ885261	MN969147	[3,27]
*T. bacillisporus*	CBS 296.48 ^T^	KM066182	AY753368	KJ885262	JF417425	[3]
*T. bohemicus*	CBS 545.86 ^T^	JN899400	KJ865719	KJ885286	JN121532	[3]
*T. boninensis*	CBS 650.95 ^T^	JN899356	KJ865721	KJ885263	KM023276	[3]
*T. borbonicus*	CBS 141340 ^T^	MG827091	MG855687	MG855688	MG855689	[20]
*T. brunneosporus*	FMR 16566 ^T^	LT962487	LT962483	LT962488	LT962485	[24]
*T. cecidicola*	CBS 101419 ^T^	AY787844	FJ753295	KJ885287	KM023309	[3]
*T. cinnabarinus*	CBS 267.72 ^T^	JN899376	AY753377	KJ885256	JN121477	[3]
*T. cinnabarinus*	CBS 357.72	–	KM066134	–	–	[3]
*T. chlamydosporus*	CBS 140635 ^T^	KU866648	KU866836	KU866732	KU866992	[5]
*T. chlorolomus*	DAOM 241016 ^T^	FJ160273	GU385736	KJ885265	KM023304	[3,27]
*T. chlorolomus*	DTO 180-F4	–	FJ753294	–	–	[3]
*T. chlorolomus*	DTO 182-A5	–	JX091597	–	–	[3]
*T. cnidii*	KACC 46617 ^T^	KF183639	KF183641	KJ885266	KM023299	[3,28]
*T. cinnabarinus*	CBS 267.72 ^T^	JN899376	AY753377	KJ885256	JN121477	[3]
*T. cinnabarinus*	CBS 357.72	–	KM066134	–	–	[3]
*T. coalescens*	CBS 103.83 ^T^	JN899366	JX091390	KJ885267	KM023277	[3]
*T. columbinus*	NRRL 58811 ^T^	KJ865739	KF196843	KJ885288	KM023270	[3]
*T. dendriticus*	CBS 660.80 ^T^	JN899339	JX091391	KF741965	KM023286	[3]
*T. dendriticus*	DAOM 226674	–	FJ753293	–	–	[3]
*T. dendriticus*	DAOM 233861	–	FJ753294	–	–	[3]
*T. derxii*	CBS 412.89 ^T^	JN899327	JX494306	KF741959	KM023282	[3,27]
*T. diversiformis*	CBS 141931 ^T^	KX961215	KX961216	KX961259	KX961274	[11]
*T. diversus*	CBS 320.48 ^T^	KJ865740	KJ865723	KJ885268	KM023285	[3]
*T. duclauxii*	CBS 322.48 ^T^	JN899342	JX091384	KF741955	JN121491	[3]
*T. emodensis*	CBS 100536 ^T^	JN899337	KJ865724	KJ885269	JF417445	[27]
*T. erythromellis*	CBS 644.80 ^T^	JN899383	HQ156945	KJ885270	KM023290	[3]
*T. euchlorocarpius*	DTO 176-I3 ^T^	AB176617	KJ865733	KJ885271	KM023303	[3]
*T. flavus*	CBS 310.38 ^T^	JN899360	JX494302	KF741949	JF417426	[3]
*T. fusiformis*	CBS 140637 ^T^	KU866656	KU866843	KU866740	KU867000	[5]
*T. georgiensis*	DI16-145 ^T^	LT558967	LT559084	–	LT795606	[12]
*T. gwangjuensis*	CNUFC WT19-1 ^T^	MK766233	MZ318448	–	MK912174	This study
*T. gwangjuensis*	CNUFC WT19-2	MK766234	MZ318449	–	MK912175	This study
*T. helicus*	CBS 335.48 ^T^	JN899359	KJ865725	KJ885289	KM023273	[3]
*T. helicus*	CBS 134.67	–	KM066133	–	–	[3]
*T. iowaense*	NRRL 66822 ^T^	MH281565	MH282578	MH282579	MH282577	[17]
*T. islandicus*	CBS 338.48 ^T^	KF984885	KF984655	KF984780	KF985018	[3]
*T. korena*	CNUFC YJW2-13 ^T^	MZ315100	MZ318450	MZ332529	MZ332533	This study
*T. korena*	CNUFC YJW2-14	MZ315101	MZ318451	MZ332530	MZ332534	This study
*T. mimosinus*	CBS 659.80 ^T^	JN899338	KJ865726	KJ885272	MN969149	[3,27]
*T. minioluteus*	CBS 642.68 ^T^	JN899346	MN969409	KJ885273	JF417443	[3]
*T. palmae*	CBS 442.88 ^T^	JN899396	HQ156947	KJ885291	KM023300	[3]
*T. piceus*	CBS 361.48 ^T^	KF984792	KF984668	KF984680	KF984899	[3]
*T. pigmentosus*	CBS 142805 ^T^	MF278330	LT855562	LT855565	LT855568	[15]
*T. pittii*	CBS 139.84 ^T^	JN899325	KJ865728	KJ885275	KM023297	[3]
*T. proteolyticus*	CBS 303.67 ^T^	JN899387	KJ865729	KJ885276	KM023301	[3]
*T. pseudostromaticus*	CBS 470.70 ^T^	JN899371	HQ156950	KJ885277	KM023298	[3]
*T. ptychoconidius*	DAOM 241017 ^T^	FJ160266	GU385733	JX140701	KM023278	[3,27]
*T. ptychoconidius*	DTO 180-E9	–	GU385734	–	–	[3]
*T. ptychoconidius*	DTO 180-F1	–	GU385735	–	–	[3]
*T. purpureogenus*	CBS 286.36 ^T^	JN899372	JX315639	KF741947	JX315709	[3,27]
*T. purpureus*	CBS 475.71 ^T^	JN899328	GU385739	KJ885292	JN121522	[3]
*T. rademirici*	CBS 140.84 ^T^	JN899386	KJ865734		KM023302	[3]
*T. radicus*	CBS 100489 ^T^	KF984878	KF984599	KF984773	KF985013	[3]
*T. ramulosus*	DAOM 241660 ^T^	EU795706	FJ753290	JX140711	KM023281	[3]
*T. ramulosus*	DTO 182-A6	–	JX091631	–	–	[3]
*T. ramulosus*	DTO 181-E3	–	JX091626	–	–	[3]
*T. ramulosus*	DTO 182-A3	–	JX091630	–	–	[3]
*T. reverso-olivaceus*	CBS 140672 ^T^	KU866646	KU866834	KU866730	KU866990	[5]
*T. rotundus*	CBS 369.48 ^T^	JN899353	KJ865730	KJ885278	KM023275	[3]
*T. rugulosus*	CBS 371.48 ^T^	KF984834	KF984575	KF984702	KF984925	[3]
*T. ryukyuensis*	NHL 2917 ^T^	AB176628	–	–	–	[3]
*T. stipitatus*	CBS 375.48 ^T^	JN899348	KM111288	KF741957	KM023280	[3]
*T. subinflatus*	CBS 652.95 ^T^	JN899397	MK450890	KJ885280	KM023308	[3,27]
*T. tabacinus*	NRRL 66727 ^T^	MG182613	MG182627	MG182606	MG182620	[17]
*T. tardifaciens*	CBS 250.94 ^T^	JN899361	KF984560	KF984682	KF984908	[27]
*T. teleomorpha*	CNUFC YJW2-5 ^T^	MZ315102	MZ318452	MZ332531	MZ332535	This study
*T. teleomorpha*	CNUFC YJW2-6	MZ315103	MZ318453	MZ332532	MZ332536	This study
*T. tenuis*	CBS 141840 ^T^	MN864275	MN863344	MN863321	MN863333	[26]
*T. trachyspermus*	CBS 373.48 ^T^	JN899354	KF114803	KJ885281	JF417432	[3]
*T. tratensis*	CBS 133146 ^T^	KF984891	KF984559	KF984690	KF984911	[3]
*T. ucrainicus*	CBS 162.67 ^T^	JN899394	KF114771	KJ885282	KM023289	[3]
*T. unicus*	CBS 100535 ^T^	JN899336	KJ865735	KJ885283	MN969150	[27]
*T. varians*	CBS 386.48 ^T^	JN899368	KJ865731	KJ885284	KM023274	[3]
*T. verruculosus*	NRRL 1050 ^T^	KF741994	KF741928	KF741944	KM023306	[27]
*T. viridulus*	CBS 252.87 ^T^	JN899314	JX091385	KF741943	JF417422	[3]
*Trichocoma paradoxa*	CBS 788.83 ^T^	JN899398	KF984556	KF984670	JN121550	[3]

CBS: Culture collection of the Westerdijk Fungal Biodiversity Institute, The Netherlands. CNUFC: Chonnam National University Fungal Collection, Gwangju, South Korea; DAOM: Agriculture Canada and Agri-Food Canada Culture Collection, Ottawa, ON, Canada; DTO: Internal Culture Collection of the CBS-Fungal Biodiversity Centre; FMR: Facultat de Medicina i Ciencies de la Salut, Reus, Spain; KACC: Korean Agricultural Culture Collection, Republic of Korea; NRRL: Agricultural Research Service Culture Collection, Peoria, IL, USA; ^T^: ex-type strain.

**Table 2:** Morphological characteristics of *Talaromyces gwangjuensis* CNUFC WT19-1 compared with those of the reference strain *Talaromyces rademirici*.

Characteristics	CNUFC WT19-1 Isolated in This Study	*Talaromyces rademirici* ^a^
Size after 7 days at 25 °C (diameter)	<1 mm on CYA	5–6 mm on CYA
	3–5 mm on YES	5–6 mm on YES
	13–15 mm on MEA	14–16 mm on MEA
	6–7 mm on OA	9–10 mm on OA
	No growth on CREA	No growth on CREA
Size after 7 days at 37 °C on CYA (diameter)	No growth	3 mm
Conidiophores	Biverticillate and monoverticillate, 39–174 × 1.5–3 µm	Biverticillate and monoverticillate; stipes smooth-walled, 25–95 × 1.5–2.5 μm; branches 10–15 μm
Metulae	Two to six, 6–10 × 1.5–2.5 µm	Two to five, divergent, 7–11 × 2–2.5 μm
Phialides	Acerose, three to eight per metula, 5.5–10 × 1.5–2 µm	Acerose, two to six per metula, 7.5–11.5 × 1.5–3 μm
Conidia	Globose, 1.5–2.0 µm, smooth-walled	Ellipsoidal, 2.5–4 × 1.5–2.5 μm, smooth
Ascomata	Absent	Absent

^a^ From the description by Yilmaz et al. [3].

**Table 3:** Morphological characteristics of *Talaromyces koreana* CNUFC YJW2-13 compared with those of the reference strains *Talaromyces boninensis* and *Talaromyces reverso-olivaceus*.

Characteristics	CNUFC YJW2-13 Isolated in This Study	*Talaromyces boninensis* ^a^	*Talaromyces reverso-olivaceus* ^b^
Size after 7 days at 25 °C (diameter)	25–28 mm on CYA	28 mm on CYA	19–23 mm on CYA
	21–24 mm on YES	NI	25–26 mm on YES
	41–45 mm on MEA	30 mm on MEA	34–37 mm on MEA
	36–39 mm on OA	32 mm on OA	33–36 mm on OA
	15–18 mm CREA	NI	No growth on CREA
Size after 7 days at 37 °C	17–19 mm on CYA	NI	18–20 mm on CYA
Conidiophores	Biverticillate, sometimes with additional branches, stipes smooth, 15–194 × 2–4 μm, branches 6–17 × 2–3 μm	Biverticillate; stipes finely rough, 25–260 × 2.5–4 μm	Biverticillate, sometimes with extra subterminal branches; stipes smooth, 50–100 × 2.5–4 μm, branches 12–15 × 2–3 μm
Metulae	Two to seven, 7.5–16 × 2–3 μm	Four to ten, 10–16(–20) × 2.5–3(–3.5) μm	Three to five, 10–13 × 3–4 μm
Phialides	Acerose, two to seven per metula, 5.5–15 × 2–3 μm	Acerose, two to six per metula, 10–15 × 2–3.5 μm	Acerose, three to five per metula, 10–12(–14) × 2.5–3 μm
Conidia	Ellipsoidal to fusiform, finely roughed, 2–3.5 × 1.5–2.5 μm	Ellipsoidal to fusiform, sometimes globose, smooth, 2–4 × 1.5–2.5 μm	Ellipsoidal to fusiform, finely roughed, 2.5–4.5 × 2.5–3 μm
Ascomata	Absent	Grayish green, globose to subglobose, 280–550 × 240–480 μm	Absent

^a^ From the description by Yilmaz et al. [3]. ^b^ From the description by Chen et al. [5]. NI: No information.

**Table 4:** Morphological characteristics of *Talaromyces teleomorpha* CNUFC YJW2-5 compared with those of the reference strain *Talaromyces helicus*.

Characteristics	CNUFC YJW2-5 Isolated in This Study	*Talaromyces helicus* ^a^
Size after 7 days at 25 °C (diameter)	26–29 mm on CYA	13–23 mm on CYA
	29–33 mm on YES	14–22 mm on YES
	45–48 mm on MEA	25–33 mm on MEA
	32–34 mm on OA	23–35 mm on OA
	1–3 on CREA	No growth on CREA
Size after 7 days at 37 °C (diameter)	15–20 mm on CYA	10–18 mm on CYA
Conidiophores	Not observed	Mono- to biverticillate, stipes smooth walled, 30–60(–80) × 2–2.5 μm
Metulae	Not observed	Two to five, 12–15 × 2–2.5 μm
Phialides	Not observed	Acerose, two to four per metula, 8.5–12(–16) × 2.5–3 μm
Conidia	Not observed	Globose to subglobose, smooth, 2.5–3.5(–4.5) × 2.2–3.5 μm
Ascomata	Creamish-white to yellow to reddish, globose to subglobose, 200–800 μm	Yellow, pastel yellow and creamish-white, globose to subglobose, 100–300 μm
Asci	Ellipsoidal, globose to subglobose, (5.5–)6.5–9 × (4.5–)6–7 μm	6–9 × 4.5–6 μm
Ascospores	Ellipsoidal, smooth, 3–4 × 2–3 μm	Ellipsoidal, smooth (some with minute spines), 2.5–4 × 2–3 μm

^a^ From the description by Yilmaz et al. [3].

## Data Availability

All sequences generated in this study were submitted to GenBank.

## Supplementary Materials

The following are available online at https://www.mdpi.com/article/10.3390/jof7090722/s1: Figure S1: Phylogram generated from the Maximum Likelihood (RAxML) analysis based on the *BenA* sequence data for species classified in *Talaromyces* section *Purpurei*. The branches with values = 100% ML BS and 1 PP are highlighted by thickened branches. The branches with values ≥ 70% ML BS and ≥ 0.95 PP indicated above or below branches. *Talaromyces tenuis* CBS 141840 was used as the outgroup. The newly generated sequences are indicated in blue. T = ex-type. Figure S2: Phylogram generated from the Maximum Likelihood (RAxML) analysis based on the *RPB2* sequence data for species classified in *Talaromyces* section *Purpurei*. The branches with values = 100% ML BS and 1 PP are highlighted by thickened branches. The branches with values ≥ 70% ML BS and ≥ 0.95 PP indicated above or below branches. *Talaromyces tenuis* CBS 141840 was used as the outgroup. The newly generated sequences are indicated in blue. T = ex-type. Figure S3: Phylogram generated from the Maximum Likelihood (RAxML) analysis based on the ITS sequence data for species classified in *Talaromyces* section *Purpurei*. The branches with values = 100% ML BS and 1 PP are highlighted by thickened branches. The branches with values ≥ 70% ML BS and ≥ 0.95 PP indicated above or below branches. *Talaromyces tenuis* CBS 141840 was used as the outgroup. The newly generated sequences are indicated in blue. T = ex-type. Figure S4: Phylogram generated from the Maximum Likelihood (RAxML) analysis based on the *BenA* sequences data for species classified in *Talaromyces* section *Helici*. The branches with values = 100% ML BS and 1 PP are highlighted by thickened branches. The branches with values ≥ 70% ML BS and ≥ 0.95 PP indicated above or below branches. *Talaromyces tenuis* CBS 141840 was used as the outgroup. The newly generated sequences are indicated in blue. T = ex-type. Figure S5: Phylogram generated from the Maximum Likelihood (RAxML) analysis based on the ITS sequences data for species classified in *Talaromyces* section *Helici*. The branches with values = 100% ML BS and 1 PP are highlighted by thickened branches. The branches with values ≥ 70% ML BS and ≥ 0.95 PP indicated above or below branches. *Talaromyces tenuis* CBS 141840 was used as the outgroup. The newly generated sequences are indicated in blue. T = ex-type. Figure S6: Phylogram generated from the Maximum Likelihood (RAxML) analysis based on the *CaM* sequence data for species classified in *Talaromyces* section *Helici*. The branches with values = 100% ML BS and 1 PP are highlighted by thickened branches. The branches with values ≥ 70% ML BS and ≥ 0.95 PP indicated above or below branches. *Talaromyces tenuis* CBS 141840 was used as the outgroup. The newly generated sequences are indicated in blue. T = ex-type. Figure S7: Phylogram generated from the Maximum Likelihood (RAxML) analysis based on the *RPB2* sequence data for species classified in *Talaromyces* section *Helici*. The branches with values = 100% ML BS and 1 PP are highlighted by thickened branches. The branches with values ≥ 70% ML BS and ≥ 0.95 PP indicated above or below branches. *Talaromyces tenuis* CBS 141840 was used as the outgroup. The newly generated sequences are indicated in blue. T = ex-type.

## References

[1] Benjamin C.R. (1995). Ascocarps of *Aspergillus* and *Penicillium*. Mycologia.

[2] Samson R.A., Yilmaz N., Houbraken J., Spierenburg H., Seifert K.A., Peterson S.W., Varga J., Frisvad J.C. (2011). Phylogeny and nomenclature of the genus *Talaromyces* and taxa accommodated in *Penicillium* subgenus *Biverticillium*. Stud. Mycol..

[3] Yilmaz N., Visagie C.M., Houbraken J., Frisvad J.C., Samson R.A. (2014). Polyphasic taxonomy of the genus *Talaromyces*. Stud. Mycol..

[4] Visagie C.M., Yilmaz N., Frisvad J.C., Houbraken J., Seifert K.A., Samson R.A., Jacobs K. (2015). Five new *Talaromyces* species with ampulliform-like phialides and globose rough walled conidia resembling *T. verruculosus*. Mycoscience.

[5] Chen A.J., Sun B.D., Houbraken J., Frisvad J.C., Yilmaz N., Zhou Y.G., Samson R.A. (2016). New *Talaromyces* species from indoor environments in China. Stud. Mycol..

[6] Luo Y., Lu X., Bi W., Liu F., Gao W. (2016). *Talaromyces rubrifaciens*, a new species discovered from heating, ventilation and air conditioning systems in China. Mycologia.

[7] Romero S.M., Romero A.I., Barrera V., Comerio R. (2016). *Talaromyces systylus*, a new synnematous species from Argentinean semiarid soil. Nova Hedwigia.

[8] Wang X.C., Chen K., Xia Y.W., Wang L., Li T., Zhuang W.Y. (2016). A new species of *Talaromyces* (Trichocomaceae) from the Xisha Islands, Hainan, China. Phytotaxa.

[9] Yilmaz N., López-Quintero C.A., Vasco-Palacios A.M., Frisvad J.C., Theelen B., Boekhout T., Samson R.A., Houbraken J. (2016). Four novel *Talaromyces* species isolated from leaf litter from Colombian Amazon rain forests. Mycol. Prog..

[10] Yilmaz N., Visagie C.M., Frisvad J.C., Houbraken J., Jacobs K., Samson R.A. (2016). Taxonomic re-evaluation of species in *Talaromyces* section *Islandici*, using a polyphasic approach. Persoonia.

[11] Crous P.W., Wingfield M.J., Burgess T.I., Carnegie A.J., Hardy G.S., Smith D., Summerell B.A., Cano-Lira J.F., Guarro J., Houbraken J. (2017). Fungal Planet description sheets 625–715. Persoonia.

[12] Guevara-Suarez M., Sutton D.A., Gené J., García D., Wiederhold N., Guarro J., Cano-Lira J.F. (2017). Four new species of *Talaromyces* from clinical sources. Mycoses.

[13] Peterson S.W., Jurjević Ž. (2017). New species of *Talaromyces* isolated from maize, indoor air, and other substrates. Mycologia.

[14] Wang X.C., Chen K., Qin W.T., Zhuang W.Y. (2017). *Talaromyces heiheensis* and *T.mangshanicus*, two new species from China. Mycol. Prog..

[15] Barbosa R.N., Bezerra J.D., Souza-Motta C.M., Frisvad J.C., Samson R.A., Oliveira N.T., Houbraken J. (2018). New *Penicillium* and *Talaromyces* species from honey, pollen and nests of stingless bees. Antonie van Leeuwenhoek.

[16] Crous P.W., Wingfield M.J., Burgess T.I., Hardy G.S., Gené J., Guarro J., Baseia I.G., García D., Gusmão L.F., Souza-Motta C.M. (2018). Fungal Planet description sheets: 716–784. Persoonia.

[17] Crous P.W., Luangsa-Ard J.J., Wingfield M.J., Carnegie A.J., Hernández-Restrepo M., Lombard L., Roux J., Barreto R.W., Baseia I.G., Cano-Lira J.F. (2018). Fungal Planet description sheets: 785–867. Persoonia.

[18] Jiang X.Z., Yu Z.D., Ruan Y.M., Wang L. (2018). Three new species of *Talaromyces* sect. *Talaromyces* discovered from soil in China. Sci. Rep..

[19] Su L., Niu Y.C. (2018). Multilocus phylogenetic analysis of *Talaromyces* species isolated from curcurbit plants in China and description of two new species, *T. curcurbitiradicus* and *T. endophyticus*. Mycologia.

[20] Varriale S., Houbraken J., Granchi Z., Pepe O., Cerullo G., Ventorino V., Chin-A-Woeng T., Meijer M., Riley R., Grigoriev I.V. (2018). *Talaromyces borbonicus* sp. nov., a novel fungus from biodegraded *Arundo donax* with potential abilities in lignocellulose conversion. Mycologia.

[21] Rajeshkumar K.C., Yilmaz N., Marathe S.D., Seifert K.A. (2019). Morphology and multigene phylogeny of *Talaromyces amyrossmaniae*, a new synnematous species belonging to the section *Trachyspermi* from India. Mycokeys.

[22] Peterson S.W., Jurjevic Z. (2019). The *Talaromyces pinophilus* species complex. Fungal Biol..

[23] Doilom M., Guo J.W., Phookamsak R., Mortimer P.E., Karunarathna S.C., Dong W., Liao C.F., Yan K., Pem D., Suwannarach N. (2020). Screening of phosphate-solubilizing fungi from air and soil in Yunnan, China: Four novel species in *Aspergillus*, *Gongronella*, *Penicillium*, and *Talaromyces*. Front. Microbiol..

[24] Rodríguez-Andrade E., Stchigel A.M., Terrab A., Guarro J., Cano-Lira J.F. (2019). Diversity of xerotolerant and xerophilic fungi in honey. IMA Fungus.

[25] Crous P.W., Cowan D.A., Maggs-Kölling G., Yilmaz N., Larsson E., Angelini C., Brandrud T.E., Dearnaley J.D., Dima B., Dovana F. (2020). Fungal Planet description sheets: 1112–1181. Persoonia.

[26] Sun B.D., Chen A.J., Houbraken J., Frisvad J.C., Wu W.P., Wei H.L., Zhou Y.G., Jiang X.Z., Samson R.A. (2020). New section and species in *Talaromyces*. Mycokeys.

[27] Houbraken J., Kocsubé S., Visagie C.M., Yilmaz N., Wang X.C., Meijer M., Kraak B., Hubka V., Bensch K., Samson R.A. (2020). Classification of *Aspergillus*, *Penicillium*, *Talaromyces* and related genera (Eurotiales): An overview of families, genera, subgenera, sections, series and species. Stud. Mycol..

[28] Sang H., An T., Kim C.S., Shin G., Sung G., Yu S.H. (2013). Two novel *Talaromyces* species isolated from medicinal crops in Korea. J. Microbiol..

[29] You Y.H., Aktaruzzaman M., Heo I., Park J.M., Hong J.W., Hong S.B. (2020). *Talaromyces halophytorum* sp. nov. isolated from roots of *Limonium tetragonum* in Korea. Mycobiology.

[30] Reyes I., Bernier L., Simard R.R. (1999). Characteristics of phosphate solubilization by an isolate of a tropical *Penicillium rugulosum* and two UV-induced mutants. FEMS Microbiol. Ecol..

[31] Narikawa T., Shinoyama H., Fujii T. (2000). A β-rutinosidase from *Penicillum rugulosum* IFO 7242 that is a peculiar flavonoid glycosidase. Biosci. Biotechnol. Biochem..

[32] Pol D., Laxman R.S., Rao M. (2012). Purification and biochemical characterization of endoglucanase from *Penicillium pinophilum* MS 20. Indian J. Biochem. Biophys..

[33] Maeda R.N., Barcelos C.A., Anna L.M.M.S. (2013). Cellulase production by *Penicillium funiculosum* and its application in the hydrolysis of sugar cane bagasse for second generation ethanol production by fed batch operation. J. Biotechnol..

[34] Yilmaz N., Houbraken J., Hoekstra E.S., Frisvad J.C., Visagie C.M., Samson R.A. (2012). Delimitation and characterisation of *Talaromyces purpurogenus* and related species. Persoonia.

[35] Frisvad J.C., Yilmaz N., Thrane U., Rasmussen K.B., Houbraken J., Samson R.A. (2013). *Talaromyces atroroseus*, a new species efficiently producing industrially relevant red pigments. PLoS ONE.

[36] Kakvan N., Heydari A., Zamanizadeh H.R., Rezaee S., Naraghi L. (2013). Development of new bioformulations using *Trichoderma* and *Talaromyces* fungal antagonists for biological control of sugar beet damping-off disease. Crop Prot.

[37] Marois J.J., Fravel D.R., Papavizas G.C. (1984). Ability of *Talaromyces flavus* to occupy the rhizosphere. Soil Biol. Biochem..

[38] Fravel D.R., Davis J.R., Sorenson L.H. (1986). Effect of *Talaromyces flavus* and metham on verticillium wilt incidence and potato yield 1984–1985. Biol. Cult. Tests.

[39] McLaren D.L., Huang H.C., Kozub G.C., Rimmer S.R. (1994). Biological control of sclerotinia wilt of sunflower with *Talaromyces flavus* and *Coniothyrium minitans*. Plant Dis..

[40] Naraghi L., Heydari A., Rezaee S., Razavi M., Jahanifar H. (2010). Study on antagonistic effects of *Talaromyces flavus* on *Verticillium albo*-*atrum*, the causal agent of potato wilt disease. Crop Prot..

[41] Pretsch A., Nag M., Schwendinger K., Kreiseder B., Wiederstein M., Pretsch D., Genov M., Hollaus R., Zinssmeister D., Debbab A. (2014). Antimicrobial and anti-inflammatory activities of endophytic fungi *Talaromyces wortmannii* extracts against acne-inducing bacteria. PLoS ONE.

[42] Deng Z.L., Ribas J.L., Gibson D.W., Connor D.H. (1988). Infections caused by *Penicillium marneffei* in China and Southeast Asia. Review of eighteen cases and report of four more Chinese cases. Rev. Infec. Dis..

[43] Hien T.V., Loc P.P., Hoa N.T.T. (2001). First case of disseminated *Penicilliosis marneffei* infection among patients with acquired immunodeficiency syndrome in Vietnam. Clin. Infect. Dis..

[44] Stolk A.C., Samson R.A. (1972). Studies on *Talaromyces* and related genera II. The genus *Talaromyces*. Stud. Mycol..

[45] Goh T.K., Hyde K.D. (1996). Biodiversity of freshwater fungi. J. Ind. Microbiol..

[46] Hawksworth D.L. (1991). The fungal dimension of biodiversity: Magnitude, significance, and conservation. Mycol. Res..

[47] Hawksworth D.L., Lücking R. (2017). Fungal diversity revisited 2.2 to 3.8 million species. Microbiol Spectr..

[48] Shearer C.A., Descals E., Kohlmeyer B., Kohlmeyer J., Marvanová L., Padgett D., Porter D., Raja H.A., Schmit J.P., Thorton H.A. (2017). Fungal biodiversity in aquatic habitats. Biodivers. Conserv..

[49] Jones E.B.G., Hyde K.D., Pang K.L. (2014). Freshwater Fungi and Fungal-Like Organisms.

[50] Nguyen T.T.T., Paul N.C., Lee H.B. (2016). Characterization of *Paecilomyces variotii* and *Talaromyces amestolkiae* in Korea based on the morphological characteristics and multigene phylogenetic analyses. Mycobiology.

[51] White T.J., Bruns T., Lee S., Taylor J., Innis M.A., Gelfand D.H., Sninsky J.J., White T.J. (1990). Amplification and direct sequencing of fungal ribosomal RNA genes for phylogenetics. PCR Protocols.

[52] Glass N.L., Donaldson G.C. (1995). Development of primer sets designed for use with the PCR to amplify conserved genes from filamentous ascomycetes. Appl. Environ. Microbiol..

[53] Peterson S.W., Vega F., Posada F., Nagai C. (2005). *Penicillium coffeae*, a new endophytic species isolated from a coffee plant and its phylogenetic relationship to *P. fellutanum*, *P. thiersii* and *P. brocae* based on parsimony analysis of multilocus DNA sequences. Mycologia.

[54] Hong S.B., Cho H.S., Shin H.D., Frisvad J.C., Samson R.A. (2006). Novel *Neosartorya* species isolated from soil in Korea. Int. J. Syst. Evol. Microbiol..

[55] Liu Y.J., Whelen S., Hall B.D. (1999). Phylogenetic relationships among ascomycetes: Evidence from an RNA polymerase II subunit. Mol. Biol. Evol..

[56] Houbraken J., Samson R.A. (2011). Phylogeny of *Penicillium* and the segregation of Trichocomaceae into three families. Stud. Mycol..

[57] Katoh K., Rozewicki J., Yamada K.D. (2019). MAFFT online service: Multiple sequence alignment, interactive sequence choice and visualization. Brief. Bioinform..

[58] Capella-Gutiérrez S., Silla-Martínez J.M., Gabaldón T. (2009). TrimAl: A tool for automated alignment trimming in large-scale phylogenetic analyses. Bioinformatics.

[59] Kumar S., Stecher G., Tamura K. (2016). MEGA7: Molecular evolutionary genetics analysis version 7.0 for bigger datasets. Mol. Biol. Evol..

[60] Glez-Peña D., Gómez-Blanco D., Reboiro-Jato M., Fdez-Riverola F., Posada D. (2010). ALTER: Program–oriented format conversion of DNA and protein alignments. Nucleic Acids. Res..

[61] Ronquist F., Teslenko M., van der Mark P., Ayres D.L., Darling A., Höhna S., Larget B., Liu L., Suchard M.A., Huelsenbeck J.P. (2012). MrBayes 3.2: Efficient Bayesian phylogenetic inference and model choice across a large model space. Syst. Biol..

[62] Rambaut A. (2009). FigTree, Version 1.3. 1. Computer Program Distributed by the Author. http://www.treebioedacuk/software/fgtree.

[63] Frisvad J.C., Thrane U. (1987). Standardized high performance liquid chromatography of 182 mycotoxins and other fungal metabolites based on alkylphenone indices and UV VIS spectra (diode array detection). J. Chromatogr..

[64] Nielsen K.F., Månsson M., Rank C., Frisvad J.C., Larsen T.O. (2011). Dereplication of microbial natural products by LC-DAD-TOFMS. J. Nat. Prod..

[65] Houbraken J., Wang L., Lee H.B., Frisvad J.C. (2016). New sections in *Penicillium* containing novel species producing patulin, pyripyropens or other bioactive compounds. Persoonia.

[66] Schoch C.L., Seifert K.A., Huhndorf S., Robert V., Spouge J.L., Levesque C.A., Chen W., Bolchacova E., Voigt K., Crous P.W. (2012). Nuclear ribosomal internal transcribed spacer (ITS) region as a universal DNA barcode marker for Fungi. Proc. Natl. Acad. Sci. USA.

[67] Yoshida E., Fujimoto H., Baba M., Yamazaki M. (1995). 4 new chlorinated azaphilones, helicusins A-D, closely related to 7-epi-sclerotiorin, from an ascomycetous fungus, *Talaromcyes helicus*. Chem. Pharm. Bull..

[68] Seifert K.A., Hoekstra E.S., Frisvad J.C., Louis-Seize G. (2004). *Penicillium cecidicola*, a new species on cynipid insect galls on *Quercus pacifica* in the western United States. Stud. Mycol..

[69] Visagie C.M., Roets F., Jacobs K. (2009). A new species of *Penicillium*, *P. ramulosum* sp. nov., from the natural environment. Mycologia.

[70] Van der Walt L., Spotts R.A., Visagie C.M., Jacobs K., Smit F.J., McLeod A. (2010). *Penicillium* species associated with preharvest wet core rot in South Africa and their pathogenicity on apple. Plant Dis..

[71] Visagie C.M., Jacobs K. (2012). Three new additions to the genus *Talaromyces* isolated from Atlantis sandveld fynbos soils. Persoonia.

[72] Heo I., Hong K., Yang H., Lee H.B., Choi Y.-J., Hong S.-B. (2019). Diversity of *Aspergillus*, *Penicillium*, and *Talaromyces* species isolated from freshwater environments in Korea. Mycobiology.

[73] Pangging M., Nguyen T.T.T., Lee H.B. (2019). New records of four species belonging to Eurotiales from soil and freshwater in Korea. Mycobiology.

[74] Visagie C.M., Houbraken J. (2020). Updating the taxonomy of *Aspergillus* in South Africa. Stud. Mycol..

