# Supplementary material for: Discovery and Extrolite Production of Three New Species of Talaromyces Belonging to Sections Helici and Purpurei from Freshwater in Korea
Source: J Fungi (Basel). 2021 Sep 3;7(9):722. doi: 10.3390/jof7090722 (PMC8471979; doi:10.3390/jof7090722)
Supplement: Supplementary file 1 [file jof-07-00722-s001.zip › jof-1269537-supplementary.pdf]

Supplementary Material

Supplementary Figures

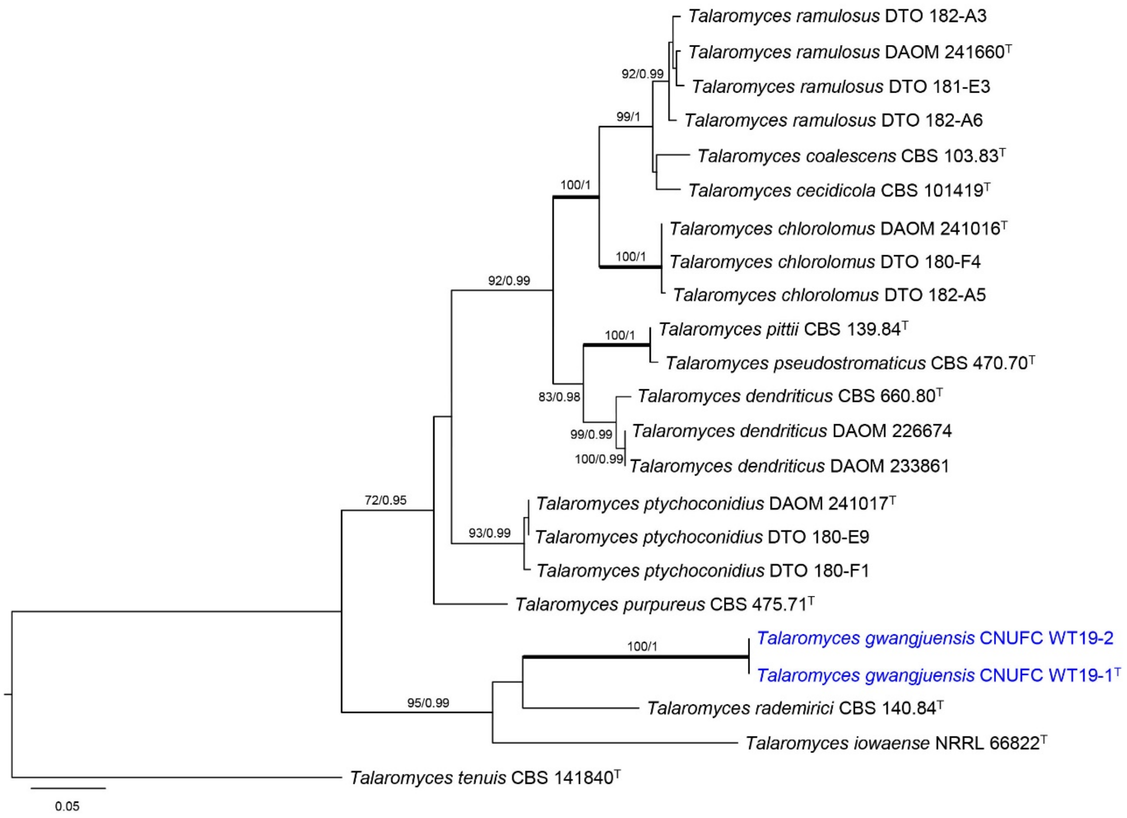

Figure S1. Phylogram generated from Maximum Likelihood (RAxML) analysis based on *BenA* sequence data for species classified in *Talaromyces* section *Purpurei*. The branches with values =100% ML BS and 1 PP are highlighted by thickened branches. The branches with values ≥ 70 % MLBS and ≥ 0.95 PP indicated above or below branches. *Talaromyces tenuis* CBS 141 840 was used as outgroup. The newly generated sequences are indicated in blue. T = ex type

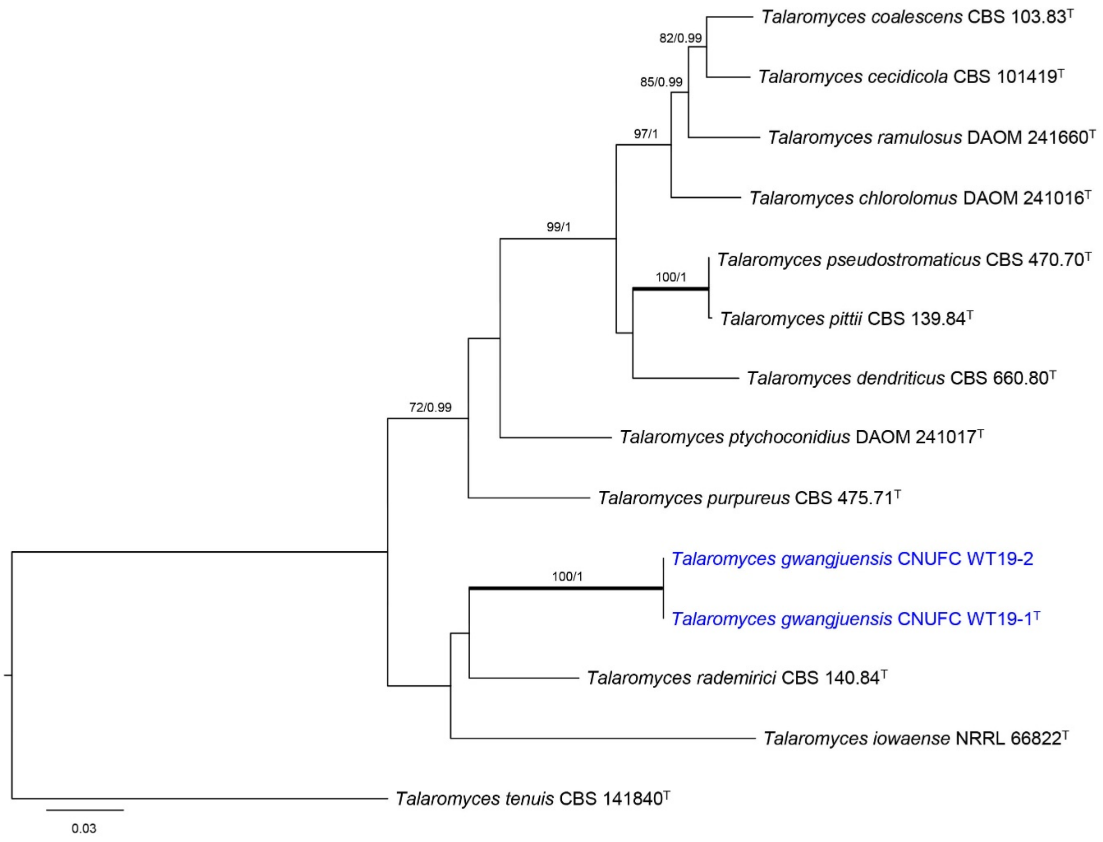

Figure S2. Phylogram generated from Maximum Likelihood (RAxML) analysis based on *RPB2* sequence data for species classified in *Talaromyces* section *Purpurei*. The branches with values =100% ML BS and 1 PP are highlighted by thickened branches. The branches with values ≥ 70 % ML BS and ≥ 0.95 PP indicated above or below branches. *Talaromyces tenuis* CBS 141840 was used as outgroup. The newly generated sequences are indicated in blue. T = ex type.

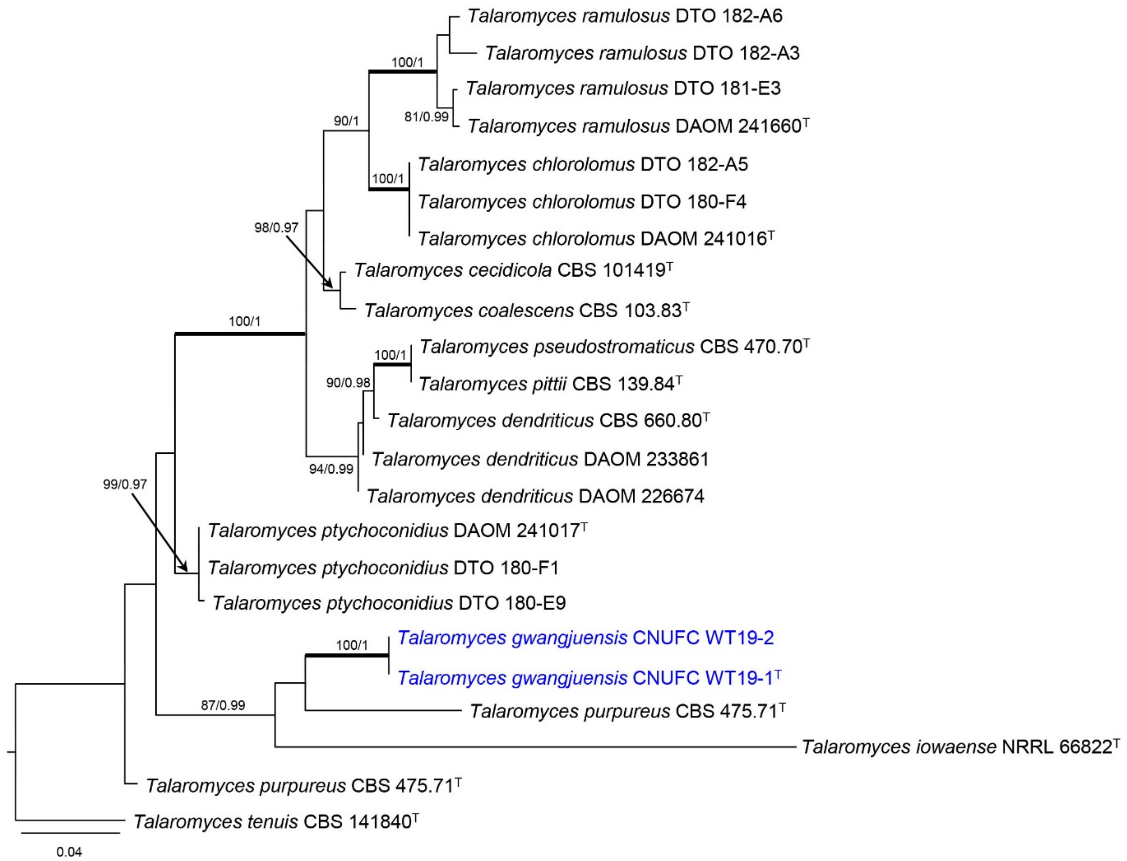

Figure S3. Phylogram generated from Maximum Likelihood (RAxML) analysis based on ITS sequence data for species classified in *Talaromyces* section *Purpurei*. The branches with values =100% ML BS and 1 PP are highlighted by thickened branches. The branches with values ≥ 70 % ML BS and ≥ 0.95 PP indicated above or below branches. *Talaromyces tenuis* CBS 141840 was used as outgroup. The newly generated sequences are indicated in green. T = ex type.

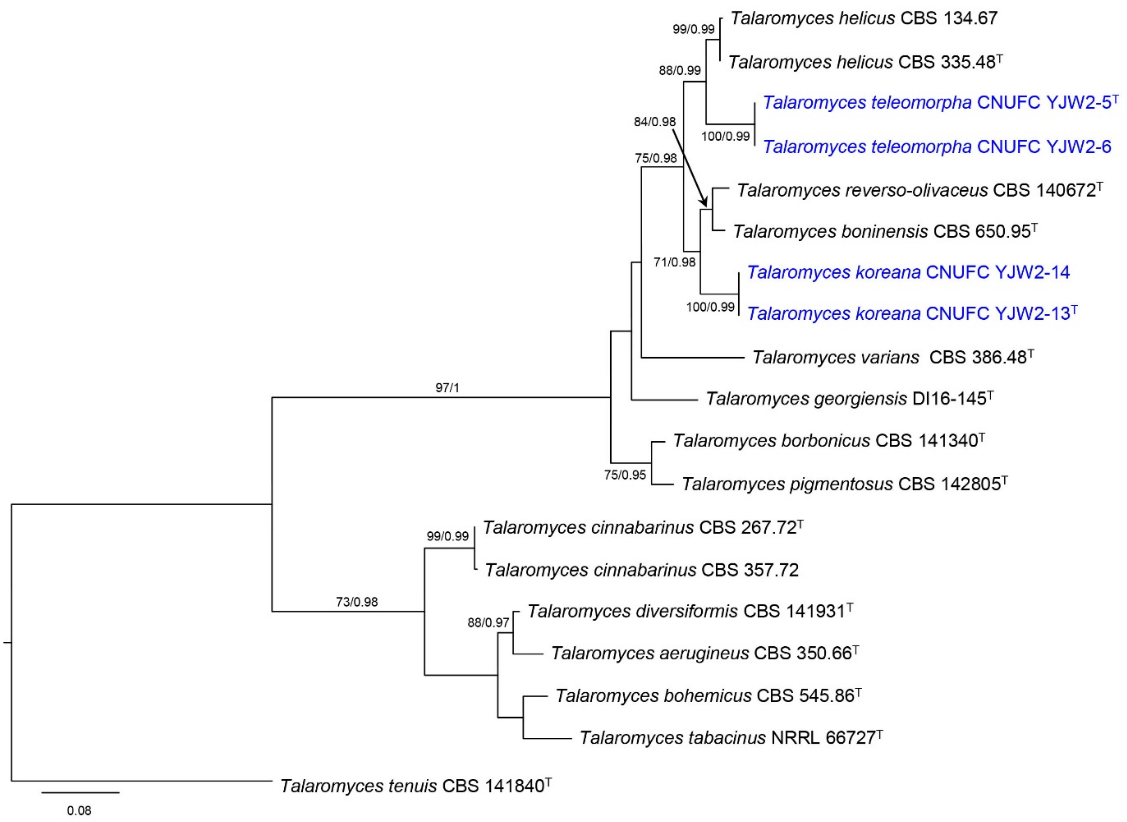

Figure S4. Phylogram generated from Maximum Likelihood (RAxML) analysis based on *BenA* sequences data for species classified in *Talaromyces* section *Helici*. The branches with values =100% ML BS and 1 PP are highlighted by thickened branches. The branches with values ≥ 70 % ML BS and ≥ 0.95 PP indicated above or below branches. *Talaromyces tenuis* CBS 141840 was used as outgroup. The newly generated sequences are indicated in blue. T = ex type.

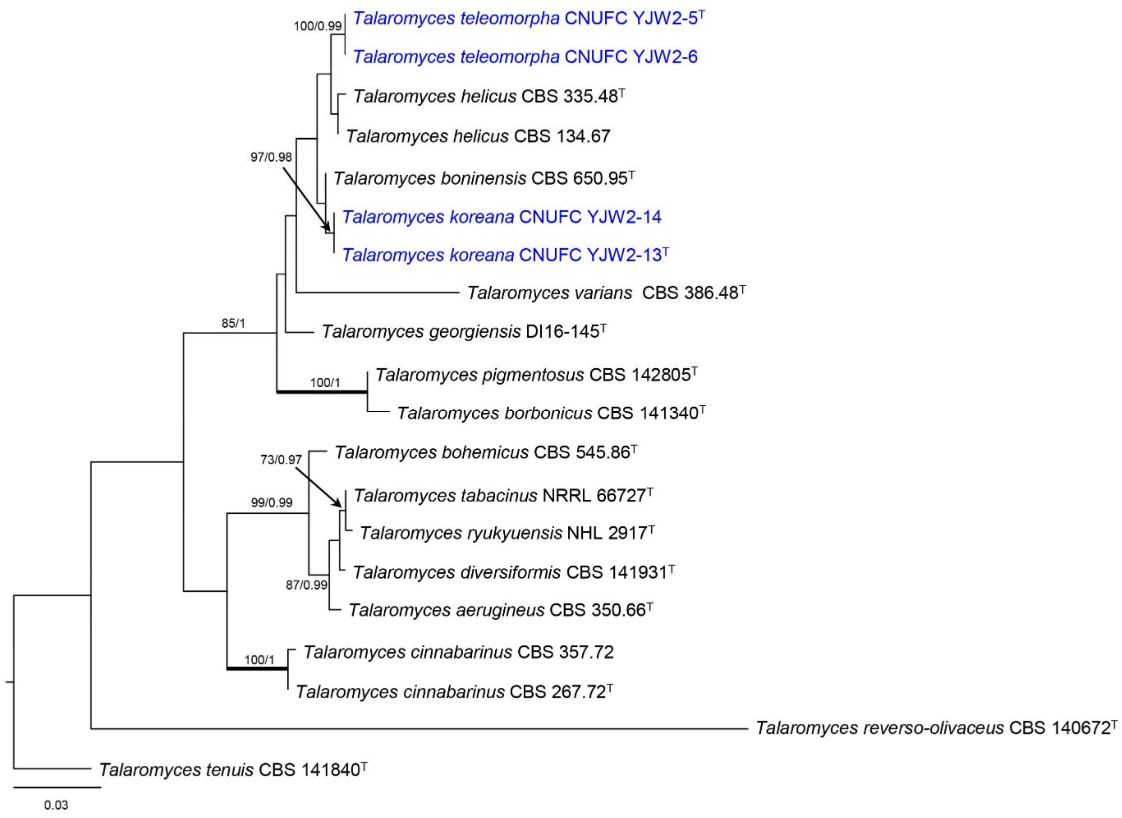

Figure S5. Phylogram generated from Maximum Likelihood (RAxML) analysis based on ITS sequence data for species classified in *Talaromyces* section *Helici*. The branches with values =100% ML BS and 1 PP are highlighted by thickened branches. The branches with values ≥ 70 % ML BS and ≥ 0.95 PP indicated above or below branches. *Talaromyces tenuis* CBS 141840 was used as outgroup. The newly generated sequences are indicated in blue. T = ex type.

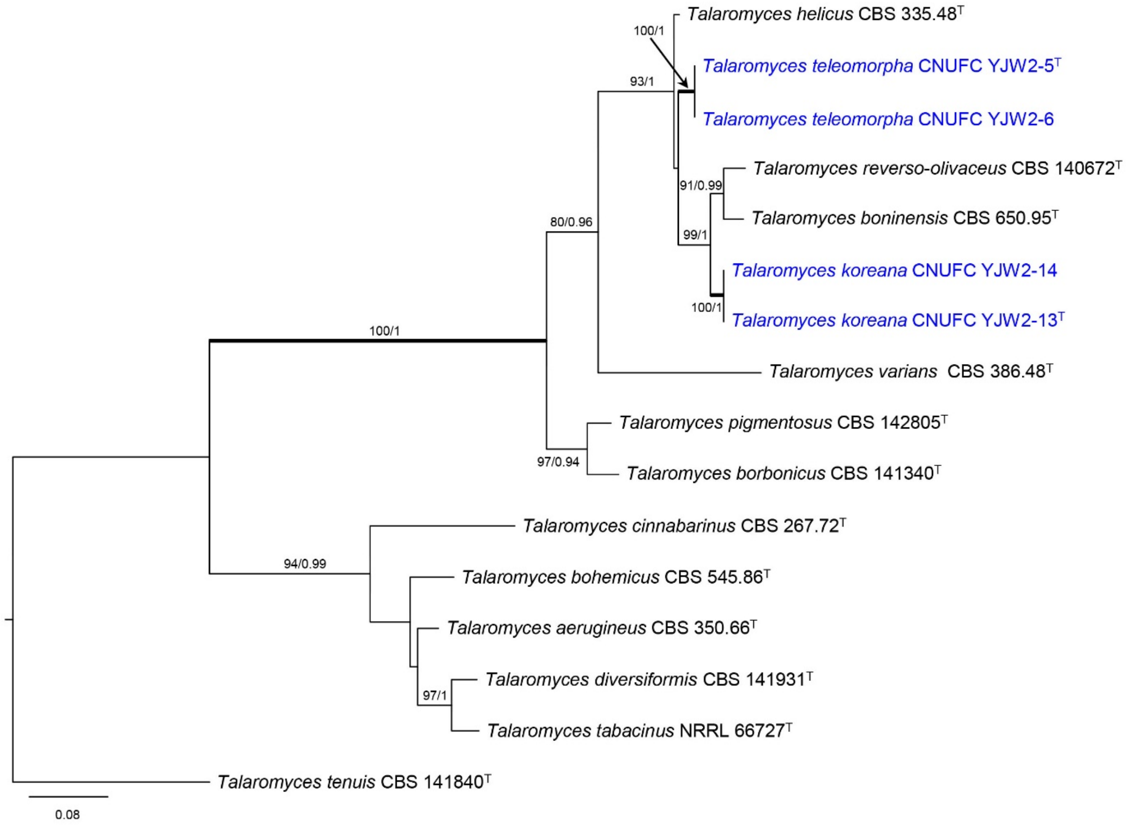

Figure S6. Phylogram generated from Maximum Likelihood (RAxML) analysis based on *CaM* sequence data for species classified in *Talaromyces* section *Helici*. The branches with values =100% ML BS and 1 PP are highlighted by thickened branches. The branches with values ≥ 70 % ML BS and ≥ 0.95 PP indicated above or below branches. *Talaromyces tenuis* CBS 141840 was used as outgroup. The newly generated sequence are indicated in blue. T = ex type.

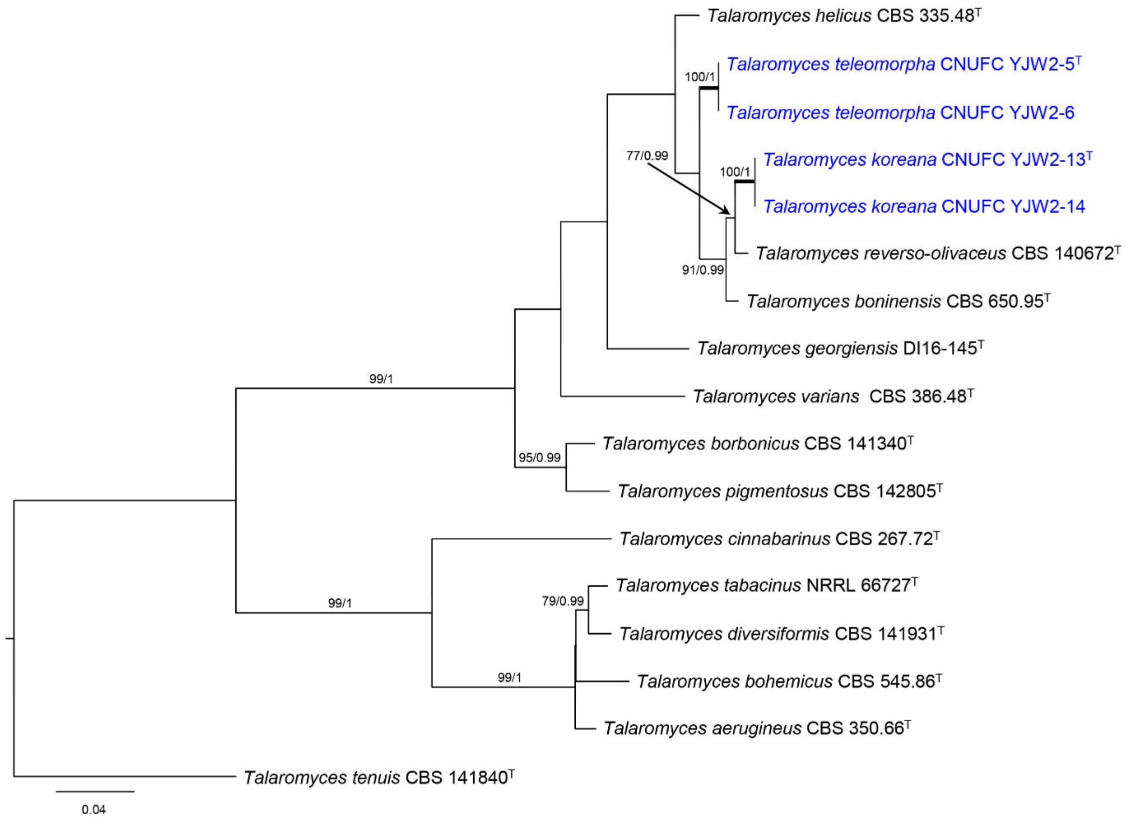

Figure S7. Phylogram generated from Maximum Likelihood (RAxML) analysis based on *RPB2* sequence data for species classified in *Talaromyces* section *Helici*. The branches with values =100% ML BS and 1 PP are highlighted by thickened branches. The branches with values ≥ 70 % ML BS and ≥ 0.95 PP indicated above or below branches. *Talaromyces tenuis* CBS 141840 was used as outgroup. The newly generated sequence are indicated in blue. T = ex type.
